# Supplementary material for: Factors Influencing Decision Making Regarding the Acceptance of the COVID-19 Vaccination in Egypt: A Cross-Sectional Study in an Urban, Well-Educated Sample
Source: Vaccines (Basel). 2021 Dec 24;10(1):20. doi: 10.3390/vaccines10010020 (PMC8780609; doi:10.3390/vaccines10010020)
Supplement: Supplementary file 1 [file vaccines-10-00020-s001.zip › vaccines-1502342-supplementary.pdf]

Factors Influencing the Acceptability of COVID-19 Vaccination in Egypt

Introduction of Research

Many countries and communities are dealing with groups and growing numbers of individuals delaying or refusing recommended vaccinations for themselves or their children. The World Health Organization has identified vaccine hesitancy and anti-vaccine attitudes as one of the top 10 health threats facing the world in 2020. This has created a need for immunization programs to find approaches and strategies to address vaccine hesitancy. The objectives of this research study are: (1) to determine the acceptability level of the COVID-19 vaccine; (2) to determine the acceptance, preferences, impact factors of future COVID-19 vaccine; and (3) to determine the influencing factors of COVID-19 vaccination acceptance between vaccine demand group and vaccine delay group. It is hoped that by understanding the factors contributing to vaccine hesitancy, headway can be made to overcome it and promote fact-based, effective public health strategies to encourage the maximum levels of vaccination.

Participation in Research, Benefits, and Risk:

Your participation in this research is entirely voluntary. You may refuse to participate in the study, or you may withdraw yourself from participation in the research any time without penalty. The study's outcome will help determine the acceptance, preferences, impact factors of future COVID-19 vaccine. Information obtained from this research will benefit researchers to analyze the acceptance level of the COVID-19 vaccine. If you would like to know the results of the study, we will notify you. Your details and response will be kept private and confidential.

How Long Will It Take?

The procedure involves filling an online survey that will take approximately 2 minutes only. Your cooperation is much needed to complete the study we are conducting. Hence, we are grateful for your participation in responding to this survey today! Feel free to forward the link to this survey and encourage your friends to answer this survey, too, as this will greatly impact our study. To contact us in regard to this research, you can email at us: roy.marzo@monash.edu

The Questionnaire

- Informed Consent

1. Do you want to participate in this survey?

Yes

No

- Section A: Socio/Demographic Profile

2. Place of residency

Rural area

Urban area

3. Age in years

....

4. Gender

Male

Female

5. Race

Egyptian

Non-Egyptian

6. Educational Level

No formal education

Primary school Secondary school

Post-secondary education (University : Matriculation / A-level, etc. / Diploma)

Tertiary education (Degree, Master, PHD)

7. Occupational Status

Full-time

Part-time

Unemployed

Student

8. Marital Status

Single

Married

Divorced

Widowed

9. Family Income

Less than 5,000

L.E. 5,000-10,000 L.E.

More than 10,000L L.E.

- Section B: The Acceptability and Factors Influencing Acceptability of

COVID-19 Vaccination

10. COVID-19 vaccination is an effective way to prevent and control COVID-19

Yes

No

11. I would like to accept vaccination if the COVID-19 vaccine is successfully developed and approved for listing in the future

Yes

No

12. Vaccine convenience (vaccination method, frequency, distance to vaccination sites) is an important factor in vaccination decision-making

Yes

No

13. Doctor’s recommendation is an important factor in vaccination decision making

Yes

No

14. Vaccine price is an important factor in vaccination decision-making

Yes

No

15. I would accept the vaccine as soon as possible if Coronavirus vaccine become available

Yes, as soon as possible

No, delay vaccination until I confirmed the vaccine safety
